# Contemplating Dichotomous Nature of Gamma Delta T Cells for Immunotherapy

**DOI:** 10.3389/fimmu.2022.894580

**Published:** 2022-05-20

**Authors:** Jaydeep Bhat, Katarzyna Placek, Simon Faissner

**Affiliations:** ^1^ Department of Molecular Immunology, Ruhr-University Bochum, Bochum, Germany; ^2^ Department of Molecular Immunology and Cell Biology, Life and Medical Sciences Institute, University of Bonn, Bonn, Germany; ^3^ Department of Neurology, Ruhr-University Bochum, St. Josef-Hospital, Bochum, Germany

**Keywords:** gamma delta T cells, immunotherapy, cancers, infection, neuroimmunology, metabolism, multi-omics

## Abstract

γδ T cells are unconventional T cells, distinguished from αβ T cells in a number of functional properties. Being small in number compared to αβ T cells, γδ T cells have surprised us with their pleiotropic roles in various diseases. γδ T cells are ambiguous in nature as they can produce a number of cytokines depending on the (micro) environmental cues and engage different immune response mechanisms, mainly due to their epigenetic plasticity. Depending on the disease condition, γδ T cells contribute to beneficial or detrimental response. In this review, we thus discuss the dichotomous nature of γδ T cells in cancer, neuroimmunology and infectious diseases. We shed light on the importance of equal consideration for systems immunology and personalized approaches, as exemplified by changes in metabolic requirements. While providing the status of immunotherapy, we will assess the metabolic (and other) considerations for better outcome of γδ T cell-based treatments.

## Introduction

T cells and B cells have emerged as primary lymphocytes lineages throughout 500 million years of evolutionary conservation, mainly generating antigen receptor diversity through somatic recombination (1, 2). The broad range of diversity is achieved by the recombination events occurring on human chromosome 7 for TCR γ and β chain genes, and on human chromosome 14 for TCR α and δ chain genes. TCR γ and δ genes in mice are located on chromosomes 13 and 14, respectively (3, 4). The variable regions of TCR chains comprising of variable (V), diversity (D), and joining (J) elements give rise to the broad range of diversity which enables recognition of foreign molecular patterns (3). Conventionally, TCR α and β chains are rearranged and expressed on the surface to become αβ T cells (~95% of CD3^+^ T cells in human peripheral blood), while TCR γ and δ chain-expressing cells become γδ T cells (~5% of CD3^+^ T cells in human peripheral blood). TCR γ and δ chain genes are further classified into subfamilies, consequently, the multiple combinations of these TCR family genes generate many functional γδ T-cell subsets such as Vγ9^+^Vδ2, Vγ9^-^Vδ2, Vδ1 and Vδ3 which can be paired with various Vγ chains. Depending on the ontogeny of the subset, the phenotypic distribution and ligand recognition change dramatically (5). The Vδ1 subset is abundant in the intestine and gut, but it is a minor population in the peripheral blood. This is in contrast to the Vδ2 subset which is a major population in circulation and a minor subset in the mucosa. The features and functions of both αβ and γδ T cells differ remarkably well depending on the thymic and extrathymic origin, as extrathymic T cells are more functionally “innate” immune cells (6, 7). Mouse γδ T-cell subsets develop through successive but coordinated waves and reside in most of the peripheral tissues. These murine γδ T cells, classified based on TCR γ chains, are mainly found in two functional states depending on interferon-γ (IFN-γ) or interleukin-17 (IL-17) production (8).

γδ T cells possess a unique potential of functional plasticity. Within the tumor milieu, γδ T cells produce cytokines (e.g. IFN-γ or IL-17), which are associated with the prognosis of different kinds of cancers. This dual roles of human γδ T cells in cancer has been recently reviewed (9). Since γδ T cells possess a dichotomous nature in cancer, autoimmunity and infections, this review will focus on mechanisms of γδ T cells in those areas; however, being aware about the fact there are important advances in other fields such as modes of antigen recognition (5, 10), fetal ontology (11, 12), and involvement in hepatic or gastro-intestinal diseases (13–16), which is out of the scope of this review. Apart from the basic understanding of γδ T-cell subsets and their function in health and diseases, the use of γδ T cells for immunotherapeutic applications is of great interest. Additionally, other recent technological advances such as single-cell omics, 3D organoid models or humanized mice will facilitate the progress in harnessing the therapeutic potential of human γδ T cells. In this review, we discuss the pivotal features of γδ T cells and their potential for therapeutic approaches.

## Dichotomy of γδ T Cells

### γδ T cells in Cancers – a Double-Edged Sword

Due to their potential of immunoserveillance and anti-tumor response, γδ T cells are found to be involved in several types of cancer including hematological malignancies (17), glioblastoma (18), gastric (19), colorectal (20) and breast cancer (21). Dysregulated mevalonate metabolism in cancer cells often leads to the accumulation of phosphoantigens (pAg) such as Isopentenyl Pyrophosphate (IPP), which potentiates Vγ9Vδ2 T-cell cytotoxicity (22). IPP can be released to the extracellular space where it is recognized by Vδ2 T cells *via* ATP-binding cassette transporter A1 (ABCA1) and apoliprotein A-I (apoA-1) (23). Recent studies have shown that phosphoantigens are bound by butyrophilins (BTN), specifically BTN3A1 and BTN2A1, which then interact with the TCR of Vδ2 T cells. Formation of such a signaling complex results in Vδ2 T-cell activation and in the anti-tumor activity (24, 25). Though the molecular details of butyrophilins- γδ TCR signaling complex is largely unknown, a landmark study showed that BTN3A1 (an isoform of CD277) and its intracellular B30.2 domain are absolutely essential for inside-out signaling to activate Vδ2 T cells (26), which is further modulated by Rho-GTPase (27). Dissecting this molecular complexity further, it was revealed that Vγ9Vδ2 TCR is required initially for T-cell activation and formation of immune synapse (IS) with CD277 (recruiting BTN3A1 and BTN2A1, independent of pAg), upon which latter provides mandatory coactivation signal and stabilizes IS in a pAg-dependent manner (28).

In addition to the butyrophilins (as mentioned above) and B7 superfamily-like proteins, major histocompatibility class (MHC) - like antigens and immunoglobulin (Ig) -like antigens have also been identified as antigens for γδ T-cell subsets [extensively reviewed in (5)]. For example, Vδ1 T cells recognize self-derived or foreign lipids bound by the CD1d molecule on the surface of target cells (29, 30). Other interesting examples of MHC-like antigens are MHC-related protein 1 (MR1), ephrin type-A receptor 2 (EphA2) and endothelial protein C receptor (EPCR) (31–33). MR1 is a Vitamin B precursor and known antigen for mucosal associated invariant T cells, but recently shown to be recognized by Vδ1 T cells from healthy individuals and in some diseases (31). EphA2 and EPCR are well known stress-ligands. EPCR serves as a ligand for human Vγ4Vδ5 subset-specific recognition of endothelial cells infected by cytomegalovirus and epithelial tumors (33). Not only EPCR, annexin A2 (an Ig-like antigen) is also recognized by Vγ8Vδ3 subset during cellular stress surveillance (34). Interestingly, non-physiological molecules like red algal protein phycoerythrin have also been reported as antigens for human and murine IL-17-producing γδ T cells (35). Furthermore, the functional plasticity of γδ T cells includes a response mediated by CD16 and thus participates in the antibody-dependent cellular cytotoxicity (ADCC). It has been shown to enhance Vδ2 T cell function towards lymphoma cells with the use of anti-CD20 (36, 37). Vγ9Vδ2 T-cell cytotoxicity can also be mediated by the production of cytokines (e.g. IFN-γ and TNF-α), cytotoxic (e.g. granzymes) and apoptotic molecules (e.g. TRAIL), and/or *via* NKG2D receptor-ligand axis (22).

γδ T cells are highly pleiotropic in function as they possess both anti-tumor and pro-tumor activities in the tumor microenvironment (TME). γδ T cells are the early producers of IFN-γ during tumorigenesis (38), while IL-2 and IL-15 are the potent inducers of cytotoxic potential (39, 40), which provide an important cancer immunomodulating factor to promote other cytotoxic T lymphocyte responses. Tumor-infiltrating γδ T cells preferentially produce IFN-γ and are positively associated with better patient outcome in case of colon cancer (20). Also, intracellular IFN-γ expression only after phorbol ester and ionomycin (PMA/Iono) stimulation was remarkable in γδ T cells from TME of ovarian cancer (41). IFN-γ producing γδ T cells exert their anti-tumor functions by upregulating MHC class I molecules and CD54, thus further enhancing CD8 T-cell-mediated killing (42). Conversely, γδ T cells producing IL-17 have been suggested to negatively impact the progression of colon (43), gallbladder (44), and breast cancer (45), either by suppressing immune cell functions, promoting immune cell pro-tumor activity, or by inducing angiogenesis. Hypoxia, which is commonly found in solid tumors, was attributed to reduce cytotoxic activity of γδ T cells in oral cancer patients (46) and enhance IL-17 production. Furthermore, γδ T cells provide pro-tumor inflammatory conditions and thus favor tumor progression, participating in most of the hallmarks of cancer (47, 48).

### γδ T Cells in Autoimmune Disease of the Central Nervous System

Classically, γδ T cells are known to possess the properties of innate immune cells such as rapid expression of IFN-γ or IL-17 in response to cytokine supplementation without TCR engagement. TME drives IL-17 production in γδ T cells and hence provide evidence for the γδ T-cell function as a consequence of (micro) environmental signals. The capacity to produce IL-17 is attributed to epigenetic regulation (49). IL-17 producing γδ T cells are implicated in autoimmunity and inflammatory conditions. Results from experimental autoimmune encephalomyelitis (EAE), a mouse model of multiple sclerosis (MS), provide evidence that γδ T cells serve as important source of cytokines IL-17 and IL-23 and consequently amplify IL-17 production by Th17 cells (50). Vice versa, IL-17A is also important for the recruitment of IL-1β secreting myeloid cells that prime pathogenic γδT17 and Th17 cells in EAE (51), suggesting a regulatory loop. One effect of IL-17 producing γδ T cells is to interfere with regulatory T cells (Treg) development by preventing the conversion of conventional T cells into Foxp3^+^ Treg cells as elicited using IL23R reporter mice (52). This observation was additionally supported by enhanced antigen-specific T cell responses by γδ T cells. In EAE, Vγ4^+^IL-17 producing γδ T cells differentiate in the draining lymph nodes, mediated by IL23R and through activation of *Il17* locus, but not *via* IL-1R1 (53).

Those alterations hold also true for MS. Using single-cell RNA-seq and spatial transcriptomics Th17/Tfh cells have been identified as cellular marker of MS disease progression (54). Though EAE is skewed towards IL-17 producing γδ T cells, studies in human have shown a more remarkable association of MS with IFN-γ producing γδ T cells. Vδ1 T cells were shown to produce a high amount of IFN-γ in newly diagnosed, untreated MS patients, which was decreased by treatment with natalizumab (55). Contrarily, single or dual expression of IFN-γ and IL-17 by Vδ2 T cells is lower in MS patients compared to healthy controls (56). There is also evidence of direct cytotoxicity towards oligodendrocytes by γδ T cells (57). γδ T cells could therefore serve as marker of disease activity. Circulating CCR5^+^ γδ T cells are decreased during MS relapse in line with higher frequency of IFN-γ^+^ γδ T cells, assuming a Th1 profile (58). Hence, beside other nonconventional immune cells, single-cell resolution identified specific γδ T cell subsets as contributor of MS disease activity with potential as therapeutic target.

### γδ T Cells in Infection

IL-17 production by Th17 cells is usually associated with protection against bacterial and fungal infection through their effector function (59). In 2009, the pivotal role of CCR6^+^ γδ T cells characterized by IL-17 production, innate receptor expression and recruitment of neutrophils was identified for the first time as first line response to mycobacteria and *Candida albicans* (60). Unlike αβ T cells, IL-17 producing γδ T cells are not associated with the engagement of TCR (50, 60, 61). These observations are highly intriguing, since transcriptionally distinct αβ-γδ co-expressing T cells have been discovered, which produce IL-17 upon stimulation by IL-1β and IL-23 and play a pathogenic role in the CNS autoimmunity in EAE. The characterization of TCR revealed that these hybrid αβ-γδ T cells are mainly Vγ4^+^ and TCRβ^+^ and importantly, provide protection against *Staphylococcus aureus* infection (62). This is consistent with findings showing that Vγ6^+^Vδ4^+^ T cells are clonally expanded in skin-draining lymph nodes after *S. aureus* infection in mice. RNA-seq analysis of TRG and TRD sequences revealed the clonal expansion of TRGV5, TRGV6, and TRDV4 (63). In contrast to murine γδ T cells, human γδ T cells play a diverse role in infection immunity. Human Vδ2 T cells have been known to respond strongly to phosphoantigens such as (E)-4-hydroxy-3-methyl-but-2-enyl pyrophosphate (HMBPP) which is a metabolite produced by microbes *via* the 2-c-methyl-D-erythritol 4-phosphate (MEP) pathway (64). Vδ2 cells are protective in *Plasmodium falciparum* infection (65). Recently, it was shown that γδ T cells kill infected red blood cells by phagocytosis and opsonization *via* CD16, in addition to the BTN3A1-TCR mediated degranulation process (66). This host defense mechanism adds a new aspect to the γδ T-cell function and engagement during immune response. Whether the phagocytotic machinery of γδ T cells is also involved during a response to other pathogens remains to be determined.

For a long time, the role of γδ T cells in mycobacterial infection has been studied in human and animal models. *Mycobacterium tuberculosis* (Mtb) was one of the first bacteria described to induce γδ T cell immune responses (67) by recognizing Mtb antigens. Bacillus Calmette-Guérin (BCG) vaccine, the only vaccine protecting against tuberculosis, has been broadly administered worldwide and it has been shown to generate a protective Vδ2 T cell memory response against Mtb infection (68). BCG has been also suggested to provide heterologous protection against infections that are not related to Mtb (69–72). In fact, in the early 90s, it was shown that *in vitro* pre-expanded γδ T cells with *M. tuberculosis* were able to proliferate in response to re-challenge with unrelated pathogens such as *Listeria monocytogenes*, group A streptococci or *S. aureus* (73). These results indicate that human γδ T cell responses are not pathogen-specific therefore raising the question, whether BCG-induced γδ T cells contribute to their cross-protective effect and whether they can develop innate immune cell memory referred to as “trained immunity”. Trained immunity was first described in monocytes and macrophages (which have a shorter half-life) (74, 75). Therefore, those might be less suitable vaccination targets to provide long-term protection. The characterization of trained immunity in long-lived γδ T cells could potentially open new avenues in designing effective vaccines with cross-protective effects. Whether immune memory responses of γδ T cells contribute to the protection induced by other vaccines still needs to be explored.

Altogether, it is crucial to broaden the mechanistic knowledge about the role of γδ T cells in infections using systems immunology approaches such as single-cell multi-omics to provide better therapeutic interventions. This is especially vital for the development of new generation vaccines, which would not only trigger αβ T cell memory responses but also harness the therapeutic potential of γδ T cells.

## γδ T Cells in Systems Immunology – a Holistic Approach

The systems approach is defined by the use of a broad strategy for understanding the outcome of a complex set of components (76). Multi-omics methods and systems immunology measures help to investigate changes in the proteome, phenome, transcriptome, epigenome, metabolome, microbiome as well as cell-to-cell communication, all of which shape immune cell responses. A decade ago, these measurements were performed on a bulk cell population. Nowadays, it is possible to use these methods at a single-cell resolution. Such datasets are made publicly available by international consortia such as ImmGen (https://www.immgen.org) for mouse immune cells and the cell atlas for humans (https://www.humancellatlas.org). Additionally, due to increasing efforts to combine interdisciplinary approaches such as computational biology together with data science and machine learning (77), a comprehensive study of immune cells and their responses is feasible in defining disease severity/progression, therapeutic response, or even vaccine effects. Indeed, a new field of “systems vaccinology” has emerged with the aim to comprehensively analyze the immune response to vaccination and understand potential new mechanisms of protection (78). This will allow immunologists to consider the individual human variation, possibly identifying the reasons driving differential immune responses. This holds specifically true for γδ T cell responses, which are largely shaped by environmental factors and not genetic control (79). A milestone study for chimeric antigen receptor (CAR) T cells by Melenhorst et al. (2022) shows an impeccable use of single-cell multi-omics and systems immunology methods (80). In this longitudinal study, authors analyzed CD19 chimeric antigen receptor (CAR) T cells in two chronic lymphocytic leukemia patients over 10 years after successful CAR T cell transfer. In the earlier time-points, CD4^-^CD8^-^Helios^hi^ CAR T cells in one of these patients were found using cytometry-by-flight (CyTOF) method. Further characterization using 5’cellular indexing of transcriptomes and epitopes by sequencing (CITE-seq) with TCR-seq found that these cells were γδ CAR T cells with specific TRDV1 and TRGV4 gene expression. However, long-term surviving CAR T cells were predominated by the CD4^+^ T cell population with cytotoxic properties especially at later time points. Yet, the origins and contribution of these cells to the remission remains to be determined. Despite a limited number of patients analyzed, this study is the first to show the potential of systems biology and single cell omics to understand the efficacy of CAR T-cell immunotherapy.

Another good example emphasizing the importance of the systems immunology approach is to evaluate the prognostic significance of immune cells in various cancers using CIBERSORT (a machine learning-based algorithm), where γδ T cells have emerged as the most favorable leukocyte with global prognostic association across 25 human cancers (81). Optimizing this computational identification approach further, tumor-infiltrating Vγ9Vδ2 T cells were variably associated with disease outcome due to considerable high inter-individual variation in its abundance (82). A combination of flow cytometry and sequencing results with the help of single sample gene set enrichment analysis (ssGSEA) method has inferred abundance of 24 immune cell types in cancer including γδ T cells. This algorithm called “Immune Cell Abundance Identifier (ImmuneCellAI)” could accurately predict response to anti-PD1 immunotherapy (83). To a limited extent, we have previously used a comprehensive approach to assess the disease progression and therapeutic response in patients with γδ T cells malignancies (84, 85). Though the transcriptome and epigenome of γδ T cells are already available, the focus is now shifted to single-cell studies (**Table 1**) as it allows to create a compendium of cell types as exemplified in mice (104) and humans (105, 106).

**Table 1:** Summary of single-cell multi-omics datasets from healthy individuals or diseases.

Study	Year	Disease	model organism	Biological source	sc-omics method
Watkin et al. (86)	2020	Allergy	Human	PBMCs from peanut allergic (PA) patients and healthy controls	scRNA-seq
Boufea et al. (87)	2020	Breast cancer	Human	peripheral blood γδ T cells from healthy adult donors and from fresh tumor biopsies of breast cancer patients	scRNA-seq
Pizzolato et al. (88)	2019	CMV	Human	PBMC and purified γδ T cells from CMV^+^ and CMV^-^ healthy donors	scRNA-seq
Jaeger et al. (89)	2021	Crohn’s disease	Human	IEL T cells sorted from two Crohn’s disease patients and two controls	scRNA-seq
10xgenomics	–	Healthy	Human	10k PBMC from a healthy donor (v3 chemistry)	scRNA-seq
Park et al. (90)	2020	Healthy	Human	dissociated cells from human thymus during development, childhood, and adult life	scRNA-seq
Tan et al. (91)	2021	Healthy	Human	γδ T cells sorted from neonatal and adult blood	scRNA-seq and paired TCR sequencing
Reitermaier et al. (92)	2021	Healthy	Human	CD3^+^ T cells FACS-sorted from single-cell suspensions of three fetal skin donors	scRNA-seq
Tan et al. (93)	2019	Healthy	Mouse	FACS-sorted Vγ6^+^ T cell, CD4^+^ and/or CD8^+^ thymocytes	scRNA-seq
Sagar et al. (94)	2020	Healthy	Mouse	Healthy fetal and adult thymus	scRNA-seq
Lee et al. (95)	2020	Healthy	Mouse	total iNKT, MAIT, and γδ T cells from the pooled thymi of BALB/c mice	scRNA-seq and paired V(D)J sequencing
Alves de Lima et al. (96)	2020	Healthy	Mouse	sorted γδ T cells from the dural meninges and spleen of 7-d-old (P7) or 8-week-old adult mice	scRNA-seq
Goldberg et al. (97)	2020	Healthy	Mouse	pan-CD45 FACS-sorted tissue-resident haematopoietic cells from white adipose tissue	scRNA-seq
Hu et al. (98)	2021	Healthy	Mouse	sorted hepatic and thymic γδ T cells	scRNA-seq
Li et al. (99)	2022	Healthy	Mouse	mouse γδ T cells from peripheral lymph nodes, spleen, and thymus	scRNA-seq and scATAC-seq
Wang et al. (100)	2021	Leukemia	Human	CD45^+^CD3^+^ cell populations from B cell-acute lymphoblastic leukemia and healthy controls	scRNA-seq and paired TCR sequencing
Melenhorst et al. (80)	2022	Leukemia	Human	sorted single CD3^+^ CAR^+^ nuclei from patient PBMC	scRNA-seq, CITE-seq and paired TCR sequencing
Gherardin et al. (101)	2021	Merkel Cell Carcinoma	Human	sorted CD3^+^ and γδ^+^ T cells from dissociated Merkel Cell Carcinomas tumor	scRNA-seq and paired TCR sequencing
Schafflick et al. (102)	2020	MS	Human	CSF and blood from MS and healthy donors	scRNA-seq
Kaufmann et al. (54)	2021	MS	Human	PBMC from MS and healthy donors	scRNA-seq
Cerapio et al. (103)	2021	Ovarian cancer	Human	γδ T-cell infiltrating lymphocytes from ovarian carcinoma	scRNA-seq

The listed datasets are generated either directly using γδ T cells or have identified γδ T cells in their computational approaches. PBMC, peripheral blood mononuclear cells; CMV, cytomegalovirus; FACS, fluorescence activated cell sorting; iNKT, invariant natural killer T cells; MAIT, mucosal associated invariant T cells; MS, multiple sclerosis; CAR, chimeric antigen receptor.

An emerging area in system immunology and single-cell methodology is to decipher the metabolic changes in γδ T cells during the development and differentiation process. Mechanistic target of rapamycin complex 1 (mTORC1) regulates a distinct metabolic requirement for thymic development of αβ and γδ T cells. Interestingly, mTORC1 signaling further coordinates developmental signals with TCR and NOTCH pathways (107). Diving into the details of metabolic requirements at a single-cell level, Single Cell ENergetIc metabolism (SCENITH) has been recently developed and used to assess γδ T cell energy metabolism (108, 109). Consequently, this study found that the metabolic requirements of IL-17^+^ γδ T cells are imprinted during early thymic development and are maintained in the periphery and tumor of obese mice (109). A distinct metabolic usage by IFN-γ^+^ versus IL-17^+^ γδ T cells shows a need for glycolysis versus oxidative metabolism, respectively. Interestingly, glucose supplementation elevated the anti-tumor function of IFN-γ^+^ γδ T cells (109). Similarly, altered tumor metabolism also needs to be studied as it is sensed by γδ T cells (32), which may ultimately implicates γδ T cell responses in TME causing hypoxia (46, 110) or tumor resistance (111). Moreover, recent reports highlight a crucial role of γδ T cells in thermogenesis and sympathetic innervation (112, 113). Furthermore, the ketogenic diet has been shown to expand protective γδ T cells during an infection with influenza virus in the lungs (114) and which restrain inflammation in adipose tissues (97). However, prolonged intake of the ketogenic diet causes obesity and significantly reduces the adipose tissue-resident γδ T cells (97). Thus, targeting metabolic changes together with transcriptional changes will help to understand the γδ T-cell differentiation process and its implications in diseases.

## γδ T Cell-Based Immunotherapy: Missing Links and Unexplored Avenues

Due to their unique characteristics distinct from conventional αβ T cells, γδ T cells are an attractive cellular target for allogeneic transfer as exemplified by the recent phase I clinical trial in 132 late-stage cancer patients (115) and ongoing clinical trial on patients with solid tumors (https://clinicaltrials.gov/ct2/show/NCT04765462). The concept of γδ T cell-based immunotherapy has been under development for more than a decade. Earlier, the immunotherapy with γδ T cells for cancer was mainly based on two approaches: *in vivo* activation of γδ T cells using aminobisphonates (e.g. zoledronate) and adoptive transfer of *in vitro* expanded γδ T cells (116). The approach of *in vivo* activation of γδ T cells has extended its toolbox. Because of the basic research on γδ T-cell activation (26), the molecular mediators of activation (e.g. butyrophilins) can be targeted to improve immunotherapy outcome. In an ongoing clinical trial, monoclonal anti-BTN3A1 antibodies (ICT01) are administered alone or in combination with the checkpoint-inhibitor pembrolizumab for hematological cancers (https://www.clinicaltrials.gov/ct2/show/NCT04243499). Besides checkpoint inhibitors (117), other immunomodulatory agents that could be used in combination with γδ T cell-based immunotherapy include epigenetic drugs (118, 119), toll-like receptor ligands (120), or even bispecific antibodies targeting Vγ9 chains of Vγ9Vδ2 T cells (121). Since most of these modulators have been proposed or shown clinical relevance *in vitro*, many are being tested in a clinical trial. Likewise, the approach of using adoptive transfer of *in vitro* expanded γδ T cells is now suggested to be supplemented with biomaterials such as cytokines [e.g. TGF-β (122)] or nutrients [e.g. Vitamin C (123)]. Furthermore, engineered cytokines have emerged as an attractive tool to improve T cell immunotherapy by modulating cell expansion, persistence, tumor homing and adaptation to TME (124). As engineered IL-2 and IL-15 are already in clinical trials, their use in γδ T cell-based therapies might also be beneficial. The use of naturally occurring nutrient supplementation such as Vitamin C could potentially be beneficial too. However, the metabolic requirement and effect of these nutrients need to be considered given the inter-individual variation in γδ T cell frequency and their subset distribution due to age, gender, and race (125). These needs can be complemented for a more personalized approach to immunotherapy to provide benefit to patients.

**Figure 1:**
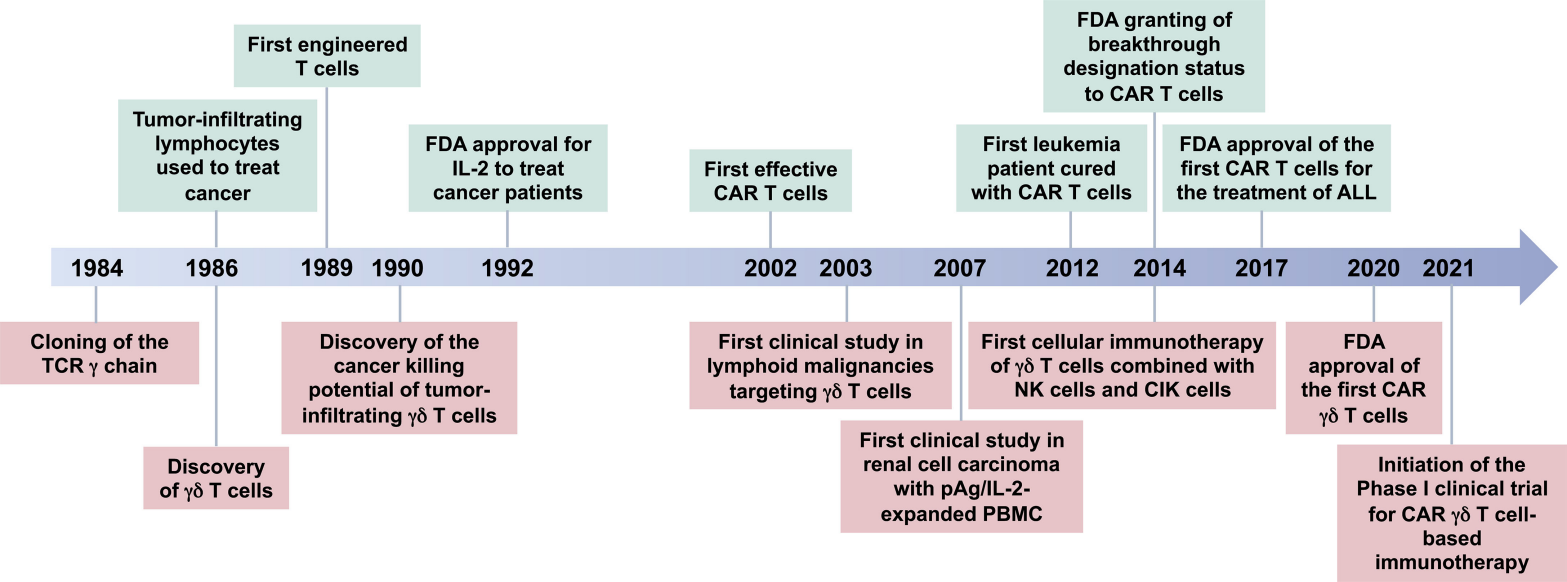
Timeline for γδ T-cell-based immunotherapy. A brief history of the breakthrough findings that led to the development of γδ T cell-based immunotherapies. γδ T cells were discovered in 1986 (126, 127), after accidental cloning of the gamma chain of the T cell receptor (TCR) in 1984 (128). At the same time, Rosenberg’s group started to treat cancer patients with their own tumor-infiltrating lymphocytes leading to the first patient to be cured from cancer using this method (129, 130). Fast forward from 1989 to year 2017 (131–137), the FDA approved the first CAR T cells for the treatment of B-cell lymphomas, Kymriah® and Yescarta® developed by Novartis Pharmaceuticals Corp. (https://www.hcp.novartis.com/home/) and Kite Pharma, Inc. (https://www.kitepharma.com/), respectively. The first big success came in 2020 for CAR therapy with γδ T cells, when the FDA cleared an investigational new drug (IND) application and orphan drug designation for GDX012 (an allogenic Vδ1 T-cell-based therapy) developed by Lymphact and later GammaDelta Therapeutics (https://gammadeltatx.com/). Also, at the same time, the Adicet Bio (https://www.adicetbio.com/) received the FDA approval for an IND application ADI-01, an allogenic CAR γδ T cell therapy targeting CD20 protein in non-Hodgkin lymphomas. In 2021, the first Phase I Clinical Trials of γδ T-cell-based immunotherapies were initiated. TCR, T-cell receptor; FDA, The United States Food and Drug Administration; IL-2, interleukin-2; CAR, chimeric antigen receptor; pAg, phosphoantigen; PBMC, peripheral blood mononuclear cells; ALL, acute lymphoblastic leukemia; NK cells, natural killer cells; CIK cells, cytokine-induced killer cells.

γδ T cell-based immunotherapy has been progressing over the last decades along with the development of T cell-based therapies (**Figure 1**). The novel first-in-class approach is being exploited to apply genetically modified γδ T cells in human (138). Previously, the Vδ1 subset of γδ T cells were designed and applied as a new cellular product called “DOT cells” for adoptive immunotherapy of leukemia patients (17, 139). Further advancing the approach of genetic modifications, CAR γδ T cell therapy is under investigation (114, 140, 141). The patient-derived xenograft model showed anti-leukemic activity and IL-15-mediated long-term persistence of CD123-CAR-DOTs in acute myeloid leukemia (142). Following the United States Food and Drug Administration (US FDA) approval for ADI-01 in the year 2020, an allogenic CAR γδ T cell therapy targeting CD20 protein was manufactured at clinical scale. As observed in a preclinical study with B-cell malignancies, CAR γδ T cell therapy generated an innate and adaptive anti-tumor immune response but no xenogeneic graft-versus-host disease (143). This CD20 CAR γδ T cell therapy is now in a phase I clinical trial (https://www.clinicaltrials.gov/ct2/show/NCT04735471). The interim analysis of the ADI-01 phase I clinical trial showed that 50% patients achieved complete response (CR) while 75% patients achieved objective response rate (ORR) without any ADI-01-related serious adverse events using CD20-CAR γδ T cell therapy (press release dated on December 6, 2021; https://www.adicetbio.com). Furthermore, the ADI-01 therapy has been just granted the Fast Track Designation by the US FDA raising hopes for even faster implementation of γδ T cell-based therapies into daily clinical practice (press release dated on April 19, 2022; https://www.adicetbio.com). CAR T cell therapy is being broadened towards T cells engineered with γδTCR (TEG) (138, 144). With approval by US FDA, genetically modified γδ T cells might benefit a broad spectrum of cancer patients. In the future, targeting γδ T cells with tailored immunotherapies might also be a potential new avenue for the treatment of other diseases such as MS and infections.

## Conclusion

Though neglected for a long time, γδ T cells have emerged as a key immune cell type, especially in cancer biology and are already investigated in clinical trials. Its pleiotropic role is further being investigated in other disorders including immune diseases such as MS, infections or transplantation. Use of “big data” and integrative multi-omics approaches enable us to more specifically unravel molecular mechanisms. This is complemented by the implementation of new methods such as 3D organoids combined with state-of-the-art technologies such as spatial transcriptomics. However, γδ T cells still require a clinical testing model for development of immunotherapy. Altogether, targeting γδ T cells will allow us to more precisely address a broad range of conditions, eventually allowing a γδ T cell targeted personalized immunotherapy.

## Author Contributions

JB and KP conceptualized and wrote the first draft of manuscript. JB, KP, and SF contributed to the discussion and made final corrections. All authors contributed to the article and approved the submitted version.

## Funding

This work was supported by the Ruhr University Bochum, Germany to JB. KP has received funding from the European Union’s Horizon 2020 research and innovation programme under the Marie Skłodowska-Curie grant agreement No. 798582 and from the Deutsche Forschungsgemeinschaft (DFG, German Research Foundation under Germany's Excellence Strategy - EXC2151 - 390873048.

## Conflict of Interest

The authors declare that the research was conducted in the absence of any commercial or financial relationships that could be construed as a potential conflict of interest.

## Publisher’s Note

All claims expressed in this article are solely those of the authors and do not necessarily represent those of their affiliated organizations, or those of the publisher, the editors and the reviewers. Any product that may be evaluated in this article, or claim that may be made by its manufacturer, is not guaranteed or endorsed by the publisher.
